# Vanadium Compounds as PTP Inhibitors

**DOI:** 10.3390/molecules22122269

**Published:** 2017-12-19

**Authors:** Elsa Irving, Andrew W. Stoker

**Affiliations:** Developmental Biology and Cancer Programme, UCL Great Ormond Street Institute of Child Health, 30 Guilford Street, London WC1N 1EH, UK; elsa.irving.15@ucl.ac.uk

**Keywords:** protein tyrosine phosphatases, PTP, vanadium, oxidovanadium, oxovanadium, vanadate, BMOV, diabetes, cancer

## Abstract

Phosphotyrosine signaling is regulated by the opposing actions of protein tyrosine kinases (PTKs) and protein tyrosine phosphatases (PTPs). Here we discuss the potential of vanadium derivatives as PTP enzyme inhibitors and metallotherapeutics. We describe how vanadate in the V oxidized state is thought to inhibit PTPs, thus acting as a pan-inhibitor of this enzyme superfamily. We discuss recent developments in the biological and biochemical actions of more complex vanadium derivatives, including decavanadate and in particular the growing number of oxidovanadium compounds with organic ligands. Pre-clinical studies involving these compounds are discussed in the anti-diabetic and anti-cancer contexts. Although in many cases PTP inhibition has been implicated, it is also clear that many such compounds have further biochemical effects in cells. There also remain concerns surrounding off-target toxicities and long-term use of vanadium compounds in vivo in humans, hindering their progress through clinical trials. Despite these current misgivings, interest in these chemicals continues and many believe they could still have therapeutic potential. If so, we argue that this field would benefit from greater focus on improving the delivery and tissue targeting of vanadium compounds in order to minimize off-target toxicities. This may then harness their full therapeutic potential.

## 1. Introduction 

In this review, we will focus on vanadium-derived chemicals as broad-specificity PTP inhibitors. We will discuss their use in pre-clinical models and look at their therapeutic potential in diseases such as cancer and diabetes. We also highlight a range of associated caveats. For those readers interested in more wide-ranging reviews of vanadium chemistry or comprehensive reviews of the growing multitude of vanadium compounds, these have been recently discussed in some other excellent reviews [1,2,3,4].

## 2. Protein Tyrosine Phosphatases (PTPs)

The successful existence of multicellular organisms relies on the ability of cells within them to communicate with one another through biochemical signalling, thus regulating responses to both intra- and extracellular stimuli. Reversible phosphorylation of tyrosine residues is a critical component of this process, and one that is itself heavily regulated. The addition and removal of phosphate groups is catalysed by two classes of enzymes, the protein tyrosine kinases (PTKs) for phosphorylation and the protein tyrosine phosphatases (PTPs) for dephosphorylation. Given the pivotal role of PTKs and PTPs in cell signalling, it is unsurprising that disruption to their normal regulation results in abnormal proliferation, survival, motility, differentiation and metabolism, underlying the pathogenesis of a range of human diseases and rendering these enzymes as promising therapeutic targets [5].

### 2.1. PTP Superfamily

**Figure 1:**
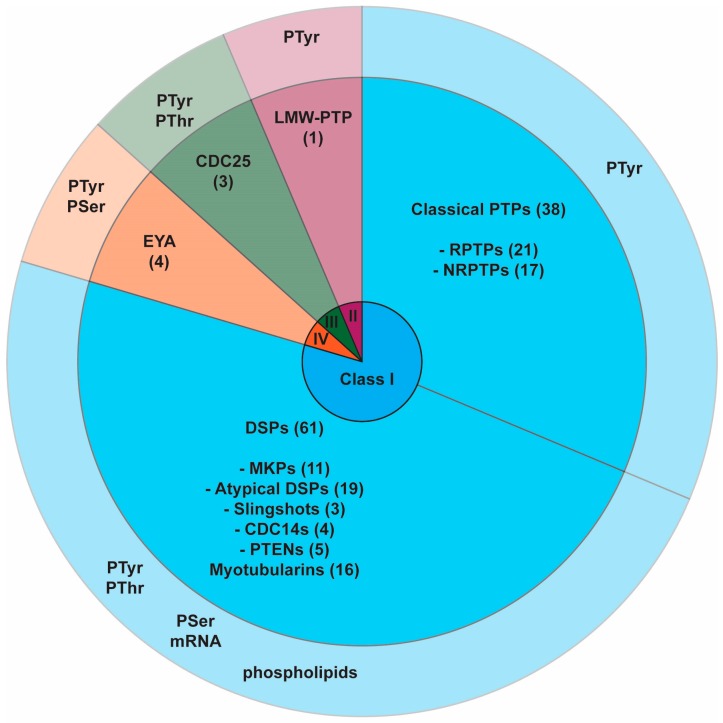
Protein tyrosine phosphatase (PTP) superfamily. Class 1-IV PTPs detailed in inner rings, with their substrate specificities stated in the outer ring.

PTPs are characterised by their active site cysteine residues, which act as critical nucleophiles during catalysis [5,6]. The PTP superfamily is made up of 107 human genes. However, of these only 81 dephosphorylate phosphotyrosine. Of the remaining 26, 13 dephosphorylate inositol phospholipids, two act on mRNA, and 11 are probably catalytically inactive [7]. They can be further subdivided into four sub-families based on the amino acid sequence of their active sites, and have differing substrate specificities (Figure 1).

### 2.2. PTP Inhibition

The roles of PTKs and PTPs in the pathogenesis of a wide range of human disease has long been appreciated and there has been significant progress in the field of kinase inhibition in the last few decades. There are over two dozen kinase inhibitors already on the market, as well as many therapeutic antibodies directed at kinases [8]. However, the development of drugs targeting specific PTPs has tended to lag behind, with no specific PTP inhibitors currently clinically approved. There are a number of potential reasons for this. First, historically there has been much greater research interest in the PTK enzyme family, with the first kinase being purified and cloned 10 years before the first PTP. Second, these kinases are often considered the main “drivers” of signalling [9,10]. There are also some technical factors that make targeting PTPs challenging. There is a high degree of sequence and structural similarity between family members, in particular at the active site containing the conserved HCX5R motif [5]. This results in difficulty in achieving a high degree of specificity when attempting to target specific PTPs [11]. Furthermore, the active sites of PTPs are much more polar than those of tyrosine kinases. Candidate inhibitor molecules that mimic substrates by competing for active site binding are thus usually charged or polar too, making them struggle to cross plasma membranes as therapeutics. However, there have been recent advances in the development of bivalent inhibitors, targeting both PTP active sites, and unique peripheral regions, which can specifically target individual PTPs [11]. For example, trodusquemine (MSI-1436) is a non-competitive allosteric inhibitor of PTP1B, currently in phase 1 clinical trials for breast cancer, and phase 2 trials for type 2 diabetes [12,13]. The general difficulty in developing specific PTP inhibitors though has commonly forced researchers in the field to use non-selective pan-inhibitors, including in particular vanadium based compounds [14].

## 3. Vanadium

As aluded to above, a common experimental strategy for PTP inhibition has been to use classes of molecules that act as pan-inhibitors of the whole enzyme family. Vanadium-derived compounds are the clearest examples of these [15]. Vanadium is a group 5d transition metal present ubiquitously in nature. It was discovered in 1830 by a Swedish chemist named Nils Gabriel Sefström and is the 18th most abundant element in the Earth’s crust, found in soil, water, air and living organisms [16]. Within many organisms vanadium is considered to be an essential micronutrient, and although this has yet to be definitively applied to humans, it is likely that vanadium plays a regulatory role in human cell function [17]. There has been considerable interest in the use of vanadium-based metallotherapeutics to treat various diseases for many decades, most notably cancer and diabetes. Since 2000 there have been over 1200 publications regarding vanadium and cancer or diabetes (PubMed). Whilst preclinical studies have and continue to provide promising data and phase I and II trials have been completed, an approved vanadium based drug has yet to reach the market. Below, we will discuss the reasons behind this current, apparent barrier to wider vanadium usage, and potential ways forward.

### 3.1. Mechanisms of PTP Inhibition 

**Figure 2:**
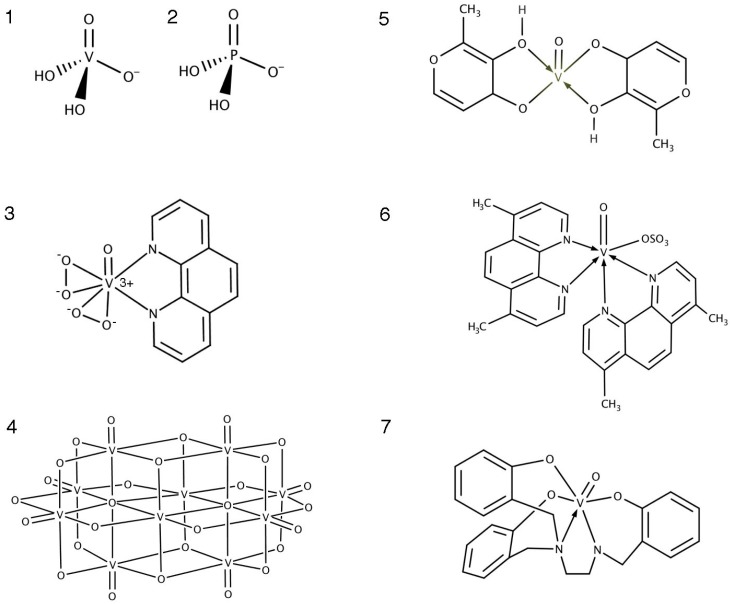
Structure **1**, orthovanadate; Structure **2**, phosphate; Structure **3**, bisperoxo(1,10-phenanthroline)oxidovanadate(V) (bpv-phen); Structure **4**, decavanadate (V10); Structure **5**, bis(maltolato)oxidovanadium (IV) (BMOV); Structure **6**, bis(4,7-dimethyl-1,10-phenanthroline)sulfatooxidovanadium(IV) (Metvan); Structure **7**, vanadium(V) oxo phenolato complex [25].

Vanadium exists in oxidation states ranging from −III to +V, however, the majority of vanadium in aqueous solutions is in the form of tetravalent vanadyl cations (IV) or pentavalent vanadate ions (V). The orthovanadate ion has a tetra-coordinated structure and a negative charge, features that it shares with the phosphate group of PTP substrates (Figure 2—structures 1 and 2). By mimicking phosphate, orthovanadate fits into the PTP active site with trigonal pyramid geometry, stabilised by a complex network of hydrogen bonds [15,18]. In this way, vanadate acts as a broad specificity, reversible and competitive PTP inhibitor [18]. Some vanadium compounds, for example the peroxidovanadium complexes, are also able to oxidise the critical active site cysteine residues, resulting in irreversible inhibition [19,20]. For example mass spectrometric analysis of the human PTP LMW-PTP and PTP1B treated with peroxidovanadium compound bisperoxo(1,10-phenanthroline)oxidovanadate(V) (bpv-phen) (Figure 1—structure 3), revealed shifts in spectra consistent with active site cysteine oxidation [15]. Note that hereafter we will refer to oxidovanadium compounds rather than oxovanadium, as the former is now recommended by IUPAC nomenclature rules. Thus where we use the prefix “oxido-”, readers need to be aware that previously published work often refers to “oxo-” for these chemicals.

Vanadium can also contribute to PTP inhibition indirectly. Interconversion between the vanadyl (IV) and vanadate (V) redox states of the vanadium ion occur in the cell via Fenton-like reactions, generating reactive oxygen species (ROS) [21,22]. These ROS can directly oxidise cysteine residues, including the critical active site cysteines in PTPs. As these cysteines are essential to the enzymatic mechanism of PTPs, their oxidation renders them catalytically inactive, sometimes irreversibly, hence contributing to broad PTP inhibition [23,24]. 

Given that PTPs share a conserved consensus sequence at the catalytic site, including the critical cysteine residue, it is reasonable to assume that vanadium compounds can inhibit all members of the PTP superfamily [14]. For example, Peters et al. showed that the oxidovanadium derivative bis(maltolato)oxidovanadium (IV) (BMOV) (Figure 2—structure 5) is able to inhibit class II LMW-PTP, as well as both receptor and non-receptor classical class I PTPs, in an in vitro enzyme/substrate competition assay [15]. Scrivens et al. also reported inhibition of dual specificity phosphatases (DSPs) and both receptor and non-receptor class I PTPs using orthovanadate and bisperoxidovanadium (bpv) compounds [20]. In relation to studies with the larger complexes generated with oxidovanadium, such as BMOV, it is very important to note that they are all likely to inhibit PTPs through orthovanadate, a common degradation product. This has various consequences for their use as metallotherapeutics and will be expanded upon below.

### 3.2. Oxidovanadium Compounds

Early examples of studies using vanadium were focussed around simple inorganic vanadium salts, whereas more recently larger, more complex compounds with organic ligands, as well as vanadium oligomers, namely decavanadate (Figure 2—structure 4), have been investigated. It is noted that distinct intracellular responses have been described for decavanadates compared with monomeric vanadium complexes, namely a distinctive oxidative stress response and an allosteric, ATPase inhibition mechanism [26,27,28,29]. However, monomeric vanadate is still released following decavanadate treatment in biological systems, and this will contribute to decavanadate-induced effects on PTPs [4,30,31].

**Figure 3:**
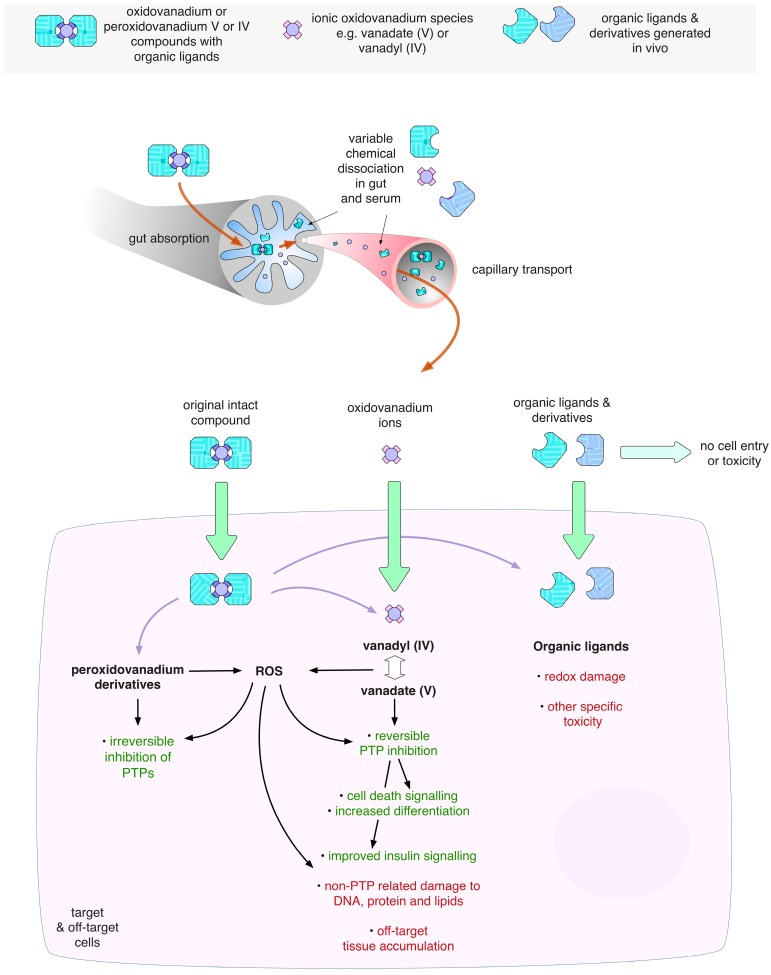
Schematic of the ingestion, chemical dissociation and cellular targeting of a generic oxidovanadium complex. Complexes of oxidovanadium with organic ligands can have good bioavailability across the gut wall, but can dissociate rapidly in the gut and blood into constituent vanadate ions and ligand derivatives. At target cells the ligands may or may not enter cells, but intracellularly they can have significant cytotoxic effects. Similarly, vanadate will enter cells and have a range of effects including PTP inhibition. Some compounds may be very stable in the blood and these may enter cells intact, where they are thought to ultimately dissociate. Oxidovanadium and peroxidovanadium compounds can generate ROS in cells indirectly or directly, respectively. Such ROS can in turn inhibit PTPs, or cause non-specific cell damage. Note that the cell shown could also represent an off-target site where these chemicals have detrimental actions.

There is an expanding repertoire of compounds containing monomeric vanadium co-ordinated by different classes of organic ligands, including but not limited to, maltolato vanadium compounds, vandocenes, and peroxidovanadium complexes [16]. One key advantage of these larger complexes with organic ligands seems to be improved bioavailability in vivo compared to vanadate [32,33]. However, increasing evidence now suggests that these larger compounds often undergo a complex range of ligand exchange events once in circulation and/or in the cell, ultimately releasing uncomplexed vanadate once again as the active PTP-inhibiting moiety [3] (Figure 3). Peters et al. reported that the NMR shifts that occur in LMW-PTP treated with either BMOV or sodium orthovanadate indicate changes in residues in the P-loop of the active site that are virtually identical to one another, indicating that the same moiety, the uncomplexed vanadium, causes the changes in both [15]. They also analysed PTP1B crystals soaked in BMOV, concluding that only the vanadate ion, and not intact BMOV, is consistent with the observed electron density.

It may therefore be true that no matter what the nature of the original vanadium complex, it may be the derived, uncomplexed vanadate that is the active PTP inhibitor. Nevertheless, the continued development of vanadium co-ordination complexes may still prove beneficial, by improving efficacy through optimised vanadate bioavailability. Indeed vanadate accumulates in various tissues at 2–3 times higher concentration following BMOV treatment compared to vanadyl sulphate [34].

### 3.3. Ligand Toxicity

With a focus on the vanadate ion, there can be a danger of losing sight of the potential, cellular actions of the organic ligands in vanadium complexes. In addition to BMOV studies (above), several further studies have shown that vanadium compounds dissociate in vitro and in circulation to release the ligating molecules, or modified forms thereof. This introduces complexity in defining how oxidovanadium complexes work in vivo, in particular if the ligands themselves have cytotoxic properties [3]. For example bis(4,7-dimethyl-1,10-phenanthroline)sulfatooxidovanadium(IV) (Metvan, Figure 2—structure 6) was shown to induce anti-cancer properties, such as ROS-associated apoptosis and loss of invasive properties, in a range of cancer cell lines within the nanomolar and low micromolar range [35,36]. However, it was also shown that the ligands themselves generate a similar toxicity profile in MTT assays of leukaemia cells [37]. Furthermore, it has now been shown that Metvan dissociates in cell culture medium to release vanadium and free 1,10-phenanthroline (phen) ligands [38,39]. Treatment with these phen ligands alone results in similar cytotoxicity in a lung cancer cell line (A549) compared to the whole Metvan complex. In this instance therefore, the tables are turned and the vanadium may be largely acting as a carrier for these toxic ligands, rather than inducing cytotoxicity itself. These ligands can also complex with copper and iron in the media, leading to enhanced cellular uptake, where they increase ROS production, ultimately leading to cell death [40]. Systemic toxicity associated with phen ligand has also been demonstrated in vivo [38,41]. Therefore, vanadium compounds containing such ligands are unlikely to be suitable for therapeutic applications directed solely at PTP enzymes themselves.

This demonstrates the importance in considering the effect of all parts of vanadium complexes in cells before prematurely assigning phenotypes as vanadium- or PTP-induced responses. In some cases therefore, the differences in biological responses to the different classes of vanadium compounds may actually be largely due to free systemic ligand effects [3]. Nevertheless, evidence indicates that some vanadium complexes, including the sixfold octahedral complex with pentadentate phenolato ligands described by Reytman et al. (Figure 2—structure 7), have a much higher stability in aqueous solution and remain intact until after they have entered the cell [25]. This particular compound displayed very high anticancer efficacy in vitro and in vivo. The pyridinone ligated oxidovanadium complexes described by Rozzo et al. are also highly stable and are likely to remain intact until inside cells where they exert anticancer activity [42]. Once inside the cell, the dynamics of complex dissociation and the relative contributions of ligand toxicity or of vanadium-induced PTP inhibition to these cellular responses is not yet known.

Overall, oxidovanadium in ligand complexes can be effective at increasing vanadium bioavailability, but caution is advised concerning the nature of the ligands used and the interpretation of biological responses.

## 4. Vanadium in Diabetes

Given its potential for modulating a wide range of signalling networks, vanadium is an attractive candidate metallotherapeutic for the treatment of a range of human disorders. In fact there has been interest in the medicinal properties of vanadium for many decades. The first reported case of vanadium use in man was by Lyonnet et al. as far back as 1899. Sixty patients, three of whom were diabetic, were given sodium metavanadate. A modest reduction in blood glucose was described for two out of the three diabetics, and no adverse effects were reported [32]. This generated some excitement surrounding the use of vanadium compounds to treat diabetic patients. However, this interest dwindled when insulin was discovered in 1922, quickly becoming the primary therapeutic for diabetes [43]. During the 1950s and 1960s vanadium compounds were in clinical trials, not for diabetes but for their ability to reduce cholesterol. Results from these trials in terms of efficacy were disappointing; however, it is worth noting that side effects were limited to nausea, abdominal pain, pharyngitis and anorexia, suggesting that vanadium compounds can be reasonably tolerated in humans [44].

In the second half of the 20th century the insulin mimetic/enhancing activity of vanadium ions began to be characterised, leading to suggestions again that vanadium compounds may indeed be able to replace insulin injections, allowing patients to be treated with less invasive oral formulations [45]. This has resulted in a new wave of research interest in developing vanadium based compounds to treat diabetes.

### 4.1. Insulin-Like Effects of Vanadium

Insulin signalling is critical for the clearance of glucose from the blood and to promote energy storage in fat cells. High levels of blood glucose trigger insulin release from the beta cells of the pancreas. Insulin binds to its cell surface receptor, inducing receptor autophosphorylation on specific tyrosine residues. These phosphotyrosines become docking sites for Src homology 2 (SH2) domain containing proteins including insulin receptor substrate 1 (IRS-1), in turn initiating signalling cascades ultimately leading to translocation of glucose transporter type 4 (GLUT-4) channels to the plasma membrane and increased glucose uptake. Insulin signalling can also promote glycogenesis, lipogenesis, and protein synthesis, whilst inhibiting lipid breakdown and gluconeogenesis. Phosphotyrosine signalling is heavily implicated throughout these signalling cascades, thus PTPs are highly relevant.

Normal insulin signalling is perturbed in diabetic patients, where either insulin can’t be produced (type 1 diabetes) or the cellular response to insulin doesn’t function correctly (type 2 diabetes). Vanadium-based compounds have resulted in reduced diabetic symptoms in animal models of type 1 and 2 diabetes, and even in some human patients [45,46,47,48,49]. This is thought to be due to the ability of vanadium to mimic insulin actions by inhibiting PTPs and in turn enhancing phosphotyrosine signalling throughout the insulin signalling network [50]. In particular, vanadium treatment increases phosphorylation of the insulin receptor, likely by inhibiting PTPs that ordinarily cause dephosphorylation at these residues [15].

### 4.2. Clinical Trials

With an increasing bank of preclinical data in favour of vanadium-based diabetes therapeutics, randomized clinical trials were carried out. Initially inorganic vanadium compounds such as vanadium sulphate and ammonium metavanadate were used [51,52,53,54,55,56]. However, these trials concluded that whilst vanadium could be tolerated, with the main adverse effect reported as minor GI distress, efficacy in diabetic patients was low and variability between patients was high [32,48]. This highlighted the need for vanadium complexes with organic ligands that would allow fine-tuning of the vanadium response. It was thought that a good ligand for vanadium complexes would prevent premature degradation of the complex, allowing sufficient time for absorption into the blood stream and reduced toxicity such as GI distress. Ideally, the complex should ultimately dissociate to leave the vanadium ion and an inert ligand to be metabolised (Figure 3). Oxidovanadium complexes such as BMOV and closely related bis(ethylmaltolato)oxidovanadium(IV) (BEOV) were thus introduced and dominated this field. In 2000 Medeval Ltd. (Manchester, UK) carried out a phase I clinical trial using BEOV administered orally in a dose escalation between 25 and 90 mg. The trial confirmed that BEOV has improved pharmacokinetics compared to vanadyl sulphate, both in terms of absorbance into circulation and increased half-life, concluding that BEOV has a bioavailability three times higher than vanadyl sulphate. There was evidence of tissue accumulation, although liver and kidney function remained normal. GI function was also normal and no other adverse effects were observed [32]. Akesis Pharmaceuticals, Inc. (La Jolla, CA, USA) subsequently completed a phase IIa using seven type 2 diabetic patients dosed once a day with 20 mg BEOV for 28 days. They reported reduced glucose levels with no major adverse effects [49].

Despite these apparently positive results, a systematic review published in 2008 stated that based on data from current clinical trials, the use of vanadium to treat diabetes could not be recommended since efficacy was too variable and side effects were consistently reported [48]. Additionally, the human vanadium trials that have been reported thus far are short term only. A longer term safety study will be required if this kind of therapy is to progress any further and potential, cumulative vanadium toxicity, in particular in high phosphate tissues such as bone, would be a major focus [34,57].

## 5. Vanadium in Cancer

### 5.1. Phosphotyrosine Signalling in Cancer

Phosphotyrosine signalling is heavily implicated in virtually all aspects of cancer biology due to its widespread influence over cell signalling. Alterations to normal phosphotyrosine signalling brought about by mutations in relevant signalling molecules can drive the initiation and progression of many different tumour types [58]. Therefore, therapeutic targeting of these pathways is an attractive avenue for cancer treatment. In the past 30 years, several tyrosine kinases have been identified as oncogenes that drive tumourigenesis, generating a rich source of druggable targets. Many small molecules and biologics targeting kinases are already in the clinic [8].

PTPs were formally discovered in the late 1980s and at that point were still thought to function as fairly unregulated housekeeping enzymes, negatively regulating phosphotyrosine signalling. They were therefore labelled as candidate tumour suppressors. Some PTPs are indeed tumour suppressors, including PTEN. PTEN is nevertheless quite specific in negatively regulating AKT signalling and it is mutated in many human malignancies [59,60]. It is now clear that many, possibly all, phosphatases are actually regulated signalling molecules that can positively contribute to the control of phosphotyrosine signalling [58]. In fact rather than simply being suppressors, MacKeigan et al. reported that nearly a third of PTPs may instead *promote* cell survival, making them potential cancer drug targets [61]. A number of oncogenic PTPs have now been described, the first of which was SHP2. Overexpression of SHP2 has been reported in leukaemia and breast cancer, and activating mutations are associated with childhood malignancies [62,63,64]. In breast cancer cell lines, shRNA-mediated inhibition of SHP2 reversed epithelial-to-mesenchymal transition and reduced migration and invasion [65]. Other PTPs such as PTP1b are also considered oncogenic in breast cancer models [66,67,68].

With the above in mind, there is now increasing interest in the development and use of PTP inhibitors for anti-cancer therapeutics. Vanadium-based chemicals may represent one source of these.

### 5.2. Anti-Cancer Activity of Vanadium

Vanadium has long been of interest in cancer biology, with the first report of its anticancer activity in 1965 [69]. Since then considerable research efforts have described the potential for vanadium-based compounds in preventing the onset of tumourigenesis and in the treatment of cancers. Vanadium compounds are able to inhibit cancer initiation and progression in model systems by acting against several of Weinberg’s hallmarks of cancer, including inducing apoptosis or other cell death pathways, reducing proliferation and inhibiting migration and metastasis [70]. The most successful cancer therapies are those that target more than one aspect of tumour biology, therefore vanadium-derived chemicals are seemingly very promising, multifunctional therapeutic candidates. In Table 1 and Table 2 we summarize several studies reporting anti-cancer properties of a variety of vanadium compounds, both in vivo and in vitro.

Many publications to date have described in vivo chemoprevention by vanadium-supplemented drinking water in chemically induced cancer models of breast, colon and liver [79,95,102,107]. These studies concluded that vanadium reduces tumour incidence, by up-regulation of drug metabolizing enzymes, protection from DNA damage, and induction of the p53 response [111] (Table 2). Moreover, various vanadium derived compounds were shown to be successful in inhibiting tumour development in xenograft models for breast cancer, hepatocellular carcinoma, glioblastoma and leukaemia, through their ability to inhibit PTPs and to induce oxidative damage, which itself likely contributes to PTP inhibition [20,35,36,72,110].

A plethora of studies using cell culture models for various tumour types have corroborated these findings and have begun to dissect the intracellular targets of vanadium compounds (Table 1). These studies highlight the fact that vanadium is able to inhibit cancer cell phenotypes via multiple nodes due to its ability to inhibit PTPs, be it directly or by ROS production, thus impacting on several key signalling pathways. In many cases, reduced proliferation and increased apoptosis is reported following vanadium treatment. For example, Ajeawung et al. reported that the vanadium compound picolinato-bis(peroxido)oxidovanadate (V) (Bpv(pic)) is able to reduce proliferation, induce apoptosis and inhibit migration in paediatric glioma cells by inhibiting activity and expression of specific PTPs involved in these processes [81]. Wu et al. reported reduced proliferation caused by G2/M cell cycle arrest in pancreatic cancer cells treated with bis(acetylacetonato)-oxidovanadium(IV), concluding that ROS-mediated ERK pathway activation is required for these anticancer effects [90]. We have shown that BMOV induces differentiation and or cytotoxicity in neuroblastoma cell lines, most likely driven at least in part by PTP inhibition [84,85]. There is also evidence that vanadium may be useful in reducing the metastatic potential of cancer cells. Petandis et al. showed that a vanadium(V)-peroxido-betaine complex can inhibit TGFβ mediated epithelial-mesenchymal transition (EMT) and reduces the tumour cell migration in vitro [77]. There is published data indicating that decavanadate-based compounds can also reduce tumour cell viability in vitro and in vivo, however, it is not clear whether this is due to PTP inhibition or other effects of these compounds (discussed below) [4,112,113].

Clearly there is a large bank of preclinical data supporting anti-cancer activities of vanadium-based compounds. Whilst many of these studies do not directly explore PTP inhibition as a mechanism driving these properties, it is likely that many of the effects described are driven by PTP inhibition, both directly by competitive inhibition by vanadate, and via ROS driven active site cysteine oxidation. If this is correct, then it is worth pointing out that the beneficial effects of vanadium compounds must be the net effect of inhibiting many PTPs.

### 5.3. Non-PTP Inhibition Mechanisms of Vanadium

Although vanadium compounds do inhibit PTPs [14], they also have other intracellular activity that may contribute to their anti-cancer efficacy. First, vanadate also affects the active site function of other phosphate-binding molecules such as ATPases, including ABC transporters [114]. Although the mechanism of this inhibition may be distinct from that of PTPs, ATPases are a class of potential off-target effectors [115]. Decavanadate also inhibits ATPases, including actin-stimulated myosin ATPase and sarcoplasmic reticulum Ca^2+^ ATPase, but in this case through a distinct, allosteric, non-competitive mechanism by binding to a site distinct from the ATP-binding pocket [4,26,27]). Decavanadate also induces specific mitochondrial effects distinct from vanadate. It accumulates in the mitochondria and alters mitochondrial antioxidant enzyme activities, as well as mitochondrial membrane depolarization perhaps by acting on complex III cytochrome *b* [4,28,29]. These findings suggest that decavanadates, like other oxidovanadium complexes, may have significant systemic toxicities if they were to be used as therapeutic compounds. Although the mitochondrial effects described above appear to be specific to decavanadate, they cannot be entirely discounted with respect to monomeric vanadium complexes as there is some evidence suggesting that decavanadate may be formed from vanadate and stabilized within cells [31,116].

As discussed previously, conversion from vanadyl to vanadate generates ROS [21]. This increase in ROS may contribute to PTP inhibition; however, it may also contribute to cell death described in some of the in vitro anti-cancer studies. Cancer cells often exist in a state of sub-lethal oxidative stress, thus even small increases in ROS may have dramatic effects on tumour cell viability by damaging DNA and lipids. Some vanadium compounds, in particular vanadocenes, can complex with DNA and inhibit RNA and DNA synthesis, likely contributing to their anticancer efficacy [117,118].

### 5.4. Systemic Toxicities Associated with Vanadium

When administered orally, vanadium enters the circulation via absorption from the GI tract. Once in the bloodstream, vanadium compounds undergo ligand exchange, and can become bound to metabolites such as lactate and citrate, and proteins, predominantly transferrin [16,119]. Vanadium can enter cells from the bloodstream via passive diffusion depending on the ligation of vanadium, active transport through anion channels and possibly by endocytosis in the case of transferrin-bound vanadium [3,120]. The relative abundance of vanadium in specific tissues is as follows; bone > kidney, liver > blood > muscle > brain [34,82]. Unabsorbed vanadium exits the body in faeces, whereas absorbed vanadium is eventually cleared in urine and from hair and skin loss. A small proportion accumulates in high phosphate tissues such as the bone for long periods of time [32,57]. As mentioned previously, this presents a potential safety concern in administering oxidovanadium as a therapeutic.

Although vanadium has not been classified by the International Agency for Research on Cancer (IARC) as a carcinogen, there have been some reports that vanadium compounds can induce tumourigenesis, potentially due to increased ROS production [19]. A study by Ress et al. sought to identify toxicity associated with long term exposure to airborne vanadium pentoxide in mice and rats, and reported increased alveolar/bronchiolar neoplasms [121]. Although, the animals in this study were exposed for two years, therefore this is perhaps not applicable to short-term oxidovanadium treatment. In very extreme cases of vanadium poisoning (6000 times higher than normal body concentration) respiration can be inhibited resulting in fatality [122]. However, generally very few adverse effects have been reported in animal models, and only GI distress, weight loss and anorexia in humans. Systemic toxicity nevertheless continues to be a major factor discouraging the use of vanadium-based compound in humans. This is clearly a greater issue for long-term use in diabetics, but perhaps less of an issue for short-term treatment of cancer patients.

## 6. Future of Vanadium Research

Vanadium-based compounds have undoubtedly displayed promising properties relating to the treatment of diabetes and cancer, as well as some other diseases [2]. However, concerns regarding their safety as therapeutic agents in humans are significant. Initially it was hoped that developing vanadium complexes with different organic ligands might reduce some of the systemic toxicity, whilst enhancing the disease-treating properties. Indeed this may still be true in the case of highly stable compounds. However, recent data demonstrate that in many cases vanadium complexes undergo speciation in circulation and in target cells, releasing vanadium and ligands, in some cases bound to other molecules [3,39]. As discussed previously, it is important then to consider the toxicity profiles of the ligands themselves, as well as systemic toxicity associated with vanadium.

One way to potentially overcome issues surrounding the off-target toxicity of ligands and oxidovanadium itself, is to package oxidovanadium compounds into nanoparticles or nanocomposites that can be passively or actively targeted to cells. For example, packaging of vanadate with chitosan into nanocomposite carriers has been demonstrated, and these have been used for diabetes studies [123,124], whereas Kremer and co-workers have successfully crosslinked oxidovanadium into complexes with anionic polysaccharides [125]. Chen et al. were able to package vanadium disulphide nanodots into PEG lipid micelles, which displayed high tumour uptake in tumour-bearing mice [126]. Although these latter nanocarriers were developed to be used for image guided photo thermal cancer therapy, this study does demonstrate the principle that vanadium compounds could be packaged into nanocarriers for delivery to tumour cells. Cancer is an appealing area for the use of nanocarriers due to the enhanced permeability and retention effect (EPR) in solid tumours, where nano-sized particles of under 200 nm are favourably delivered to and accumulate in tumour tissue due to its increased vasculature permeability [127,128]. It may even be feasible to employ V2O5 nanocrystals in this way, as they too can be formed into 100 nM particles [129], although it is unclear how these would behave in vivo. By reducing off-target, systemic toxicity of vanadium, one could better harness vanadium’s anti-PTP actions. Encapsulating drug complexes also means that degradation in the circulation can be reduced, further concentrating the amount of active complex reaching the target tissue. If deliberately used in this manner, one could envisage the design of bi-potential oxidovanadium complexes whereby cells would be subjected to both the anti-cancer effects of the PTP-inhibitory vanadate plus the cytotoxic ligands, generating additive or synergistic anti-cancer effects (Figure 3).

In summary, although there are significant caveats hindering the use of current vanadium-based therapeutics, there are good reasons why this field should maintain its efforts. With new technologies for drug delivery constantly becoming available, several of the less advantageous effects of vanadium could become avoidable, raising the opportunity for development of truly effective and safe drugs based on this transition metal and its attractive, flexible chemistry. 

## Figures and Tables

**Table 1:** Summary of some reported anti-cancer activities of vanadium in cancer cell lines.

Tumour Cell Type	Compound	Effect	Suggested Mechanism
Cervical [71]	Nicotinoyl hydrazine vanadium complexes (50–100 μM)	Increased apoptosis	p53 induction
Hepatocellular carcinoma (HCC) [72]	Sodium orthovanadate (15–30 μM)	Decreased Proliferation G2/M arrest Increased Apoptosis	
Osteosarcoma [73,74]	Oxidovanadium flavonoid complexes (10–100 μM)	Increased apoptosis DNA damage Cell cycle arrest	ROS production DNA strand breaks
Osteosarcoma [75]	Vanadium (IV) complexes (2.5–5 μM)	Reduced cell adhesion and migration Reduced colony formation	Reduced actin polymerisation via suppressed PKA activity
Malignant melanoma [42]	Pyridinone ligated oxidovanadium complexes (1–100 μM)	Reduced proliferation Increased apoptosis Cell cycle arrest	
Lung and Melanoma [76]	Pyridoxylideneiminato vanadium (50 μM)	Increased apoptosis	ROS production
Lung and breast [77]	Vanadium (V)-peroxido-betaine (25–50 μM)	Reduced migration Increased cell death	Reduced TGFβ mediated EMT
Lung and Breast [78]	Vanadium-peroxido-betaine (100–400 μM)	Increased apoptosis DNA damage	ROS production Reduced HRAS and MMP2 expression
Breast [79]	Ammonium monovanadate (100–250 μM)	Apoptosis and cell cycle arrest	
Breast [80]	Vanadocene dichloride (10–20 μM)	Reduced proliferation G2/M arrest	
Glioma [81]	Picolinato-bis(peroxido)oxidovanadate (V) (Bpv(pic)) (5–20 μM)	Reduced proliferation S phase and G2/M accumulation Increased apoptosis Reduced migration and invasion	Inhibition of PTP expression and activity
Rhabdomyosarcoma [82]	BMOV and vanadium salts (10–40 μM)	Growth inhibition	
CML [83]	VO-salen (6–32 μM)	Reduced proliferation G2/M arrest Chemosensitisation to taxol	
Neuroblastoma [84,85]	BMOV (10 μM)	Cytotoxicity Differentiation	PTP inhibition
Testicular [86]	Vanadocene dichloride (100 μM)	Apoptosis	
Prostate [87]	Vanadate (25–100 μM)	G2/M arrest Growth inhibition	ROS mediated CDC25C degradation
Ovarian and Prostate [88]	Heteroleptic Schiff base vanadium complexes (1–150 μM)	Cytotoxicity	Disrupted mitotic spindle formation
Pancreas [89]	Phenanthroline/quinolone ligated vanadium (1–100 μM)	Increased apoptosis and necroptosis G2/M arrest	ROS production
Pancreas [90]	Bis(acetylacetonato)-oxidovanadium (IV) (1–400 μM)	Reduced proliferation G2/M arrest	ROS production ERK pathway activation
Colorectal [91]	Schiff base vanadium complex (20 μg/mL)	Increased apoptosis G2/M arrest	GSH depletion ROS production DNA damage

**Table 2:** Summary of some reported anti-cancer activities of vanadium in animal cancer models.

Model	Compound	Effect	Suggested Mechanism
DEN rat liver model [92,93,94,95,96,97]	Ammonium metavanadate (0.5 ppm/4.27 μM in drinking water)	Chemopreventative—reduced proliferation and premalignant nodule incidence	Reduced DNA damage Increased expression of drug metabolising enzymes
2-AAF rat liver model [98,99,100]	Ammonium monovanadate/ammonium metavanadate (0.5 ppm/4.27 μM in drinking water)	Chemopreventative—reduced tumour incidence, reduced proliferation and increased apoptosis	Reduced DNA damage Increased expression of drug metabolising enzymes Induction of p53
Orthotopic Hepatocellular carcinoma mouse model [72]	Sodium orthovanadate (10–20 mg/kg)	Reduced cell proliferation and tumour volume	
DMH rat colon model [101,102]	Vanadium (0.5 ppm/4.27 μM in drinking water)	Chemopreventative—reduced proliferation, increased apoptosis	Reduced DNA damage Induction of p53
DMH rat colon model [103]	Ammonium monovanadate (0.5 ppm/4.27 μM in drinking water)	Chemopreventative—reduced tumour incidence	Reduced DNA damage
MNU rat mammary model [104]	Vanadyl sulphate (25 ppm in feed)	Chemopreventative—reduced tumour incidence and increase survival	
DMBA rat mammary model [79,105,106,107]	Ammonium monovanadate/ammonium metavanadate (0.5 ppm/4.27 μM in drinking water)	Chemopreventative—reduced tumour incidence and size. Reduced proliferation and increased apoptosis	Reduced DNA damage Induction of p53
DMBA rat mammary model [108]	Ammonium monovanadate (0.5 ppm/4.27 μM in drinking water)	Chemopreventative—reduced tumour incidence, reduced proliferation and increased apoptosis	Reduced DNA damage Induction of p53
DMBA rat mammary model [109]	Ammonium monovanadate (0.5 ppm/4.27 μM in drinking water)	Chemopreventative—reduced tumour incidence	Increased expression of drug metabolising enzymes
MDA-MB-231 mouse breast cancer xenograft model [35,36]	Metvan (10 mg/kg intraperitoneal)	Reduced tumour progression and increased apoptosis	Induction of oxidative damage
DA3 mouse breast cancer xenograft model [20]	Bisperoxidovanadium compounds (20 mg/kg intraperitoneal)	Reduced tumour growth	CDC25A inhibition leading to cell cycle arrest and apoptosis
U87 mouse glioblastoma xenograft model [35,36]	Metvan (10 mg/kg intraperitoneal)	Reduced tumour progression and increased apoptosis	Induction of oxidative damage
L1210 injected mice (leukemia) [110]	Vanadocene dichloride (10–130 mg/kg)	Increased life span	

## References

[1] Pessoa J.C. (2015). Thirty years through vanadium chemistry. J. Inorg. Biochem..

[2] Rehder D. (2016). Perspectives for vanadium in health issues. Future Med. Chem..

[3] Levina A., Lay P.A. (2017). Stabilities and biological activities of vanadium drugs: What is the nature of the active species?. Chem. Asian J..

[4] Aureliano M. (2016). Decavanadate toxicology and pharmacological activities: V10 or V1, both or none?. Oxid. Med. Cell. Longev..

[5] Tonks N.K. (2006). Protein tyrosine phosphatases: From genes, to function, to disease. Nat. Rev. Mol. Cell Biol..

[6] Guan K.L., Dixon J.E. (1991). Evidence for protein-tyrosine-phosphatase catalysis proceeding via a cysteine-phosphate intermediate. J. Biol. Chem..

[7] Alonso A., Sasin J., Bottini N., Friedberg I., Osterman A., Godzik A., Hunter T., Dixon J., Mustelin T. (2004). Protein tyrosine phosphatases in the human genome. Cell.

[8] Wu P., Nielsen T.E., Clausen M.H. (2016). Small-molecule kinase inhibitors: An analysis of fda-approved drugs. Drug Discov. Today.

[9] Charbonneau H., Tonks N.K., Kumar S., Diltz C.D., Harrylock M., Cool D.E., Krebs E.G., Fischer E.H., Walsh K.A. (1989). Human placenta protein-tyrosine-phosphatase: Amino acid sequence and relationship to a family of receptor-like proteins. Proc. Natl. Acad. Sci. USA.

[10] Guan K.L., Haun R.S., Watson S.J., Geahlen R.L., Dixon J.E. (1990). Cloning and expression of a protein-tyrosine-phosphatase. Proc. Natl. Acad. Sci. USA.

[11] Zhang Z.Y. (2017). Drugging the undruggable: Therapeutic potential of targeting protein tyrosine phosphatases. Acc. Chem. Res..

[12] Krishnan N., Koveal D., Miller D.H., Xue B., Akshinthala S.D., Kragelj J., Jensen M.R., Gauss C.M., Page R., Blackledge M. (2014). Targeting the disordered c terminus of PTP1B with an allosteric inhibitor. Nat. Chem. Biol..

[13] Thompson D., Morrice N., Grant L., Le Sommer S., Lees E.K., Mody N., Wilson H.M., Delibegovic M. (2017). Pharmacological inhibition of protein tyrosine phosphatase 1B protects against atherosclerotic plaque formation in the LDLR^-/-^ mouse model of atherosclerosis. Clin. Sci. (Lond.).

[14] Gordon J.A. (1991). Use of vanadate as protein-phosphotyrosine phosphatase inhibitor. Methods Enzymol..

[15] Peters K.G., Davis M.G., Howard B.W., Pokross M., Rastogi V., Diven C., Greis K.D., Eby-Wilkens E., Maier M., Evdokimov A. (2003). Mechanism of insulin sensitization by BMOV (bis maltolato oxo vanadium); unliganded vanadium (VO4) as the active component. J. Inorg. Biochem..

[16] Rehder D. (2012). The potentiality of vanadium in medicinal applications. Future Med Chem.

[17] Schroeder H.A., Balassa J.J., Tipton I.H. (1963). Abnormal trace metals in man—Vanadium. J. Chronic Dis..

[18] Crans D.C., Smee J.J., Gaidamauskas E., Yang L. (2004). The chemistry and biochemistry of vanadium and the biological activities exerted by vanadium compounds. Chem. Rev..

[19] Evangelou A.M. (2002). Vanadium in cancer treatment. Crit. Rev. Oncol. Hematol..

[20] Scrivens P.J., Alaoui-Jamali M.A., Giannini G., Wang T., Loignon M., Batist G., Sandor V.A. (2003). Cdc25a-inhibitory properties and antineoplastic activity of bisperoxovanadium analogues. Mol. Cancer Ther..

[21] Nechay B.R. (1984). Mechanisms of action of vanadium. Annu. Rev. Pharmacol. Toxicol..

[22] Sakurai H., Shimomura S., Fukuzawa K., Ishizu K. (1980). Detection of oxovanadium (IV) and characterization of its ligand environment in subcellular fractions of the liver of rats treated with pentavalent vanadium(V). Biochem. Biophys. Res. Commun..

[23] Ostman A., Frijhoff J., Sandin A., Böhmer F.D. (2011). Regulation of protein tyrosine phosphatases by reversible oxidation. J. Biochem..

[24] Barford D. (2004). The role of cysteine residues as redox-sensitive regulatory switches. Curr. Opin. Struct. Biol..

[25] Reytman L., Braitbard O., Hochman J., Tshuva E.Y. (2016). Highly effective and hydrolytically stable vanadium(V) amino phenolato antitumor agents. Inorg. Chem..

[26] Tiago T., Martel P., Gutierrez-Merino C., Aureliano M. (2007). Binding modes of decavanadate to myosin and inhibition of the actomyosin atpase activity. Biochim. Biophys. Acta.

[27] Fraqueza G., Batista de Carvalho L.A., Marques M.P., Maia L., Ohlin C.A., Casey W.H., Aureliano M. (2012). Decavanadate, decaniobate, tungstate and molybdate interactions with sarcoplasmic reticulum Ca^2+^-atpase: Quercetin prevents cysteine oxidation by vanadate but does not reverse atpase inhibition. Dalton Trans..

[28] Soares S.S., Gutierrez-Merino C., Aureliano M. (2007). Decavanadate induces mitochondrial membrane depolarization and inhibits oxygen consumption. J. Inorg. Biochem..

[29] Soares S.S., Gutierrez-Merino C., Aureliano M. (2007). Mitochondria as a target for decavanadate toxicity in sparus aurata heart. Aquat. Toxicol..

[30] Aureliano M., Gandara R.M. (2005). Decavanadate effects in biological systems. J. Inorg. Biochem..

[31] Aureliano M., Crans D.C. (2009). Decavanadate (V_10_O_28_^6−^) and oxovanadates: Oxometalates with many biological activities. J. Inorg. Biochem..

[32] Thompson K.H., Orvig C. (2006). Vanadium in diabetes: 100 years from phase 0 to phase I. J. Inorg. Biochem..

[33] McNeill J.H., Yuen V.G., Hoveyda H.R., Orvig C. (1992). Bis(maltolato)oxovanadium(IV) is a potent insulin mimic. J. Med. Chem..

[34] Setyawati I.A., Thompson K.H., Yuen V.G., Sun Y., Battell M., Lyster D.M., Vo C., Ruth T.J., Zeisler S., McNeill J.H. (1998). Kinetic analysis and comparison of uptake, distribution, and excretion of ^48^V-labeled compounds in rats. J. Appl. Physiol. (1985).

[35] D’Cruz O.J., Uckun F.M. (2002). Metvan: A novel oxovanadium(IV) complex with broad spectrum anticancer activity. Expert Opin. Investig. Drugs.

[36] Narla R.K., Chen C.L., Dong Y., Uckun F.M. (2001). In vivo antitumor activity of bis(4,7-dimethyl-1,10-phenanthroline) sulfatooxovanadium(Iv) (METVAN [VO(SO4)(Me2-Phen)2]). Clin. Cancer Res..

[37] Dong Y., Narla R.K., Sudbeck E., Uckun F.M. (2000). Synthesis, X-ray structure, and anti-leukemic activity of oxovanadium(IV) complexes. J. Inorg. Biochem..

[38] Le M., Rathje O., Levina A., Lay P.A. (2017). High cytotoxicity of vanadium(IV) complexes with 1,10-phenanthroline and related ligands is due to decomposition in cell culture medium. J. Biol. Inorg. Chem..

[39] Sanna D., Ugone V., Micera G., Buglyó P., Bíró L., Garribba E. (2017). Speciation in human blood of metvan, a vanadium based potential anti-tumor drug. Dalton Trans..

[40] Coyle B., Kinsella P., McCann M., Devereux M., O’Connor R., Clynes M., Kavanagh K. (2004). Induction of apoptosis in yeast and mammalian cells by exposure to 1,10-phenanthroline metal complexes. Toxicol. In Vitro.

[41] Li S., Crooks P.A., Wei X., de Leon J. (2004). Toxicity of dipyridyl compounds and related compounds. Crit. Rev. Toxicol..

[42] Rozzo C., Sanna D., Garribba E., Serra M., Cantara A., Palmieri G., Pisano M. (2017). Antitumoral effect of vanadium compounds in malignant melanoma cell lines. J. Inorg. Biochem..

[43] Banting F.G., Best C.H., Collip J.B., Campbell W.R., Fletcher A.A. (1922). Pancreatic extracts in the treatment of diabetes mellitus. Can. Med. Assoc. J..

[44] Somerville J., Davies B. (1962). Effect of vanadium on serum cholesterol. Am. Heart J..

[45] Heyliger C.E., Tahiliani A.G., McNeill J.H. (1985). Effect of vanadate on elevated blood glucose and depressed cardiac performance of diabetic rats. Science.

[46] Poucheret P., Verma S., Grynpas M.D., McNeill J.H. (1998). Vanadium and diabetes. Mol. Cell. Biochem..

[47] Srivastava A.K., Mehdi M.Z. (2005). Insulino-mimetic and anti-diabetic effects of vanadium compounds. Diabet. Med..

[48] Smith D.M., Pickering R.M., Lewith G.T. (2008). A systematic review of vanadium oral supplements for glycaemic control in type 2 diabetes mellitus. QJM.

[49] Thompson K.H., Lichter J., LeBel C., Scaife M.C., McNeill J.H., Orvig C. (2009). Vanadium treatment of type 2 diabetes: A view to the future. J. Inorg. Biochem..

[50] Mehdi M.Z., Pandey S.K., Théberge J.F., Srivastava A.K. (2006). Insulin signal mimicry as a mechanism for the insulin-like effects of vanadium. Cell Biochem. Biophys..

[51] Goldfine A.B., Simonson D.C., Folli F., Patti M.E., Kahn C.R. (1995). Metabolic effects of sodium metavanadate in humans with insulin-dependent and noninsulin-dependent diabetes mellitus in vivo and in vitro studies. J. Clin. Endocrinol. Metab..

[52] Goldfine A.B., Patti M.E., Zuberi L., Goldstein B.J., LeBlanc R., Landaker E.J., Jiang Z.Y., Willsky G.R., Kahn C.R. (2000). Metabolic effects of vanadyl sulfate in humans with non-insulin-dependent diabetes mellitus: In vivo and in vitro studies. Metabolism.

[53] Cohen N., Halberstam M., Shlimovich P., Chang C.J., Shamoon H., Rossetti L. (1995). Oral vanadyl sulfate improves hepatic and peripheral insulin sensitivity in patients with non-insulin-dependent diabetes mellitus. J. Clin. Investig..

[54] Halberstam M., Cohen N., Shlimovich P., Rossetti L., Shamoon H. (1996). Oral vanadyl sulfate improves insulin sensitivity in niddm but not in obese nondiabetic subjects. Diabetes.

[55] Boden G., Chen X., Ruiz J., van Rossum G.D., Turco S. (1996). Effects of vanadyl sulfate on carbohydrate and lipid metabolism in patients with non-insulin-dependent diabetes mellitus. Metabolism.

[56] Cusi K., Cukier S., DeFronzo R.A., Torres M., Puchulu F.M., Redondo J.C. (2001). Vanadyl sulfate improves hepatic and muscle insulin sensitivity in type 2 diabetes. J. Clin. Endocrinol. Metab..

[57] Domingo J.L. (2000). Vanadium and diabetes. What about vanadium toxicity?. Mol. Cell. Biochem..

[58] He R.J., Yu Z.H., Zhang R.Y., Zhang Z.Y. (2014). Protein tyrosine phosphatases as potential therapeutic targets. Acta Pharmacol. Sin..

[59] Hollander M.C., Blumenthal G.M., Dennis P.A. (2011). Pten loss in the continuum of common cancers, rare syndromes and mouse models. Nat. Rev. Cancer.

[60] Song M.S., Salmena L., Pandolfi P.P. (2012). The functions and regulation of the pten tumour suppressor. Nat. Rev. Mol. Cell Biol..

[61] MacKeigan J.P., Murphy L.O., Blenis J. (2005). Sensitized RNAi screen of human kinases and phosphatases identifies new regulators of apoptosis and chemoresistance. Nat. Cell Biol..

[62] Zhou X., Coad J., Ducatman B., Agazie Y.M. (2008). Shp2 is up-regulated in breast cancer cells and in infiltrating ductal carcinoma of the breast, implying its involvement in breast oncogenesis. Histopathology.

[63] Xu R., Yu Y., Zheng S., Zhao X., Dong Q., He Z., Liang Y., Lu Q., Fang Y., Gan X. (2005). Overexpression of Shp2 tyrosine phosphatase is implicated in leukemogenesis in adult human leukemia. Blood.

[64] Bentires-Alj M., Paez J.G., David F.S., Keilhack H., Halmos B., Naoki K., Maris J.M., Richardson A., Bardelli A., Sugarbaker D.J. (2004). Activating mutations of the noonan syndrome-associated SHP2/PTPN11 gene in human solid tumors and adult acute myelogenous leukemia. Cancer Res..

[65] Zhou X.D., Agazie Y.M. (2008). Inhibition of SHP2 leads to mesenchymal to epithelial transition in breast cancer cells. Cell Death Differ..

[66] Julien S.G., Dubé N., Read M., Penney J., Paquet M., Han Y., Kennedy B.P., Muller W.J., Tremblay M.L. (2007). Protein tyrosine phosphatase 1B deficiency or inhibition delays ErbB2-induced mammary tumorigenesis and protects from lung metastasis. Nat. Genet..

[67] Julien S.G., Dubé N., Hardy S., Tremblay M.L. (2011). Inside the human cancer tyrosine phosphatome. Nat. Rev. Cancer.

[68] Elson A. (2017). Stepping out of the shadows: Oncogenic and tumor-promoting protein tyrosine phosphatases. Int. J. Biochem. Cell Biol..

[69] Kieler J., Gromek A., Nissen N.I. (1965). Studies on the antineoplastic effect of vanadium salts. Acta Chir. Scand. Suppl..

[70] Hanahan D., Weinberg R.A. (2011). Hallmarks of cancer: The next generation. Cell.

[71] Nair R.S., Kuriakose M., Somasundaram V., Shenoi V., Kurup M.R., Srinivas P. (2014). The molecular response of vanadium complexes of nicotinoyl hydrazone in cervical cancers—A possible interference with hpv oncogenic markers. Life Sci..

[72] Wu Y., Ma Y., Xu Z., Wang D., Zhao B., Pan H., Wang J., Xu D., Zhao X., Pan S. (2014). Sodium orthovanadate inhibits growth of human hepatocellular carcinoma cells in vitro and in an orthotopic model in vivo. Cancer Lett..

[73] Leon I.E., Di Virgilio A.L., Porro V., Muglia C.I., Naso L.G., Williams P.A., Bollati-Fogolin M., Etcheverry S.B. (2013). Antitumor properties of a vanadyl(IV) complex with the flavonoid chrysin [VO(chrysin)2EtOH]2 in a human osteosarcoma model: The role of oxidative stress and apoptosis. Dalton Trans..

[74] Leon I.E., Porro V., Di Virgilio A.L., Naso L.G., Williams P.A., Bollati-Fogolín M., Etcheverry S.B. (2014). Antiproliferative and apoptosis-inducing activity of an oxidovanadium(IV) complex with the flavonoid silibinin against osteosarcoma cells. J. Biol. Inorg. Chem..

[75] Molinuevo M.S., Cortizo A.M., Etcheverry S.B. (2008). Vanadium(IV) complexes inhibit adhesion, migration and colony formation of UMR106 osteosarcoma cells. Cancer Chemother. Pharmacol..

[76] Strianese M., Basile A., Mazzone A., Morello S., Turco M.C., Pellecchia C. (2013). Therapeutic potential of a pyridoxal-based vanadium(IV) complex showing selective cytotoxicity for cancer versus healthy cells. J. Cell. Physiol..

[77] Petanidis S., Kioseoglou E., Domvri K., Zarogoulidis P., Carthy J.M., Anestakis D., Moustakas A., Salifoglou A. (2016). In vitro and ex vivo vanadium antitumor activity in (TGF-β)-induced emt. Synergistic activity with carboplatin and correlation with tumor metastasis in cancer patients. Int. J. Biochem. Cell Biol..

[78] Petanidis S., Kioseoglou E., Hadzopoulou-Cladaras M., Salifoglou A. (2013). Novel ternary vanadium-betaine-peroxido species suppresses H-ras and matrix metalloproteinase-2 expression by increasing reactive oxygen species-mediated apoptosis in cancer cells. Cancer Lett..

[79] Ray R.S., Ghosh B., Rana A., Chatterjee M. (2007). Suppression of cell proliferation, induction of apoptosis and cell cycle arrest: Chemopreventive activity of vanadium in vivo and in vitro. Int. J. Cancer.

[80] Navara C.S., Benyumov A., Vassilev A., Narla R.K., Ghosh P., Uckun F.M. (2001). Vanadocenes as potent anti-proliferative agents disrupting mitotic spindle formation in cancer cells. Anticancer Drugs.

[81] Ajeawung N.F., Faure R., Jones C., Kamnasaran D. (2013). Preclinical evaluation of dipotassium bisperoxo (picolinato) oxovanadate v for the treatment of pediatric low-grade gliomas. Future Oncol..

[82] Dąbroś W., Adamczyk A., Ciurkot K., Kordowiak A.M. (2011). Vanadium compounds affect growth and morphology of human rhabdomyosarcoma cell line. Pol. J. Pathol..

[83] Meshkini A., Yazdanparast R. (2010). Chemosensitization of human leukemia K562 cells to taxol by a vanadium-salen complex. Exp. Mol. Pathol..

[84] Clark O., Daga S., Stoker A.W. (2013). Tyrosine phosphatase inhibitors combined with retinoic acid can enhance differentiation of neuroblastoma cells and trigger ERK- and AKT-dependent, p53-independent senescence. Cancer Lett..

[85] Clark O., Park I., Di Florio A., Cichon A.C., Rustin S., Jugov R., Maeshima R., Stoker A.W. (2015). Oxovanadium-based inhibitors can drive redox-sensitive cytotoxicity in neuroblastoma cells and synergise strongly with buthionine sulfoximine. Cancer Lett..

[86] Ghosh P., D’Cruz O.J., Narla R.K., Uckun F.M. (2000). Apoptosis-inducing vanadocene compounds against human testicular cancer. Clin. Cancer Res..

[87] Liu T.T., Liu Y.J., Wang Q., Yang X.G., Wang K. (2012). Reactive-oxygen-species-mediated Cdc25C degradation results in differential antiproliferative activities of vanadate, tungstate, and molybdate in the PC-3 human prostate cancer cell line. J. Biol. Inorg. Chem..

[88] Scalese G., Mosquillo M.F., Rostán S., Castiglioni J., Alho I., Pérez L., Correia I., Marques F., Costa Pessoa J., Gambino D. (2017). Heteroleptic oxidovanadium(IV) complexes of 2-hydroxynaphtylaldimine and polypyridyl ligands against trypanosoma cruzi and prostate cancer cells. J. Inorg. Biochem..

[89] Kowalski S., Hać S., Wyrzykowski D., Zauszkiewicz-Pawlak A., Inkielewicz-Stępniak I. (2017). Selective cytotoxicity of vanadium complexes on human pancreatic ductal adenocarcinoma cell line by inducing necroptosis, apoptosis and mitotic catastrophe process. Oncotarget.

[90] Wu J.X., Hong Y.H., Yang X.G. (2016). Bis(acetylacetonato)-oxidovanadium(IV) and sodium metavanadate inhibit cell proliferation via ROS-induced sustained MAPK/ERK activation but with elevated AKT activity in human pancreatic cancer AsPC-1 cells. J. Biol. Inorg. Chem..

[91] Sinha A., Banerjee K., Banerjee A., Sarkar A., Ahir M., Adhikary A., Chatterjee M., Choudhuri S.K. (2017). Induction of apoptosis in human colorectal cancer cell line, HCT-116 by a vanadium- schiff base complex. Biomed. Pharmacother..

[92] Chakraborty T., Swamy A.H., Chatterjee A., Rana B., Shyamsundar A., Chatterjee M. (2007). Molecular basis of vanadium-mediated inhibition of hepatocellular preneoplasia during experimental hepatocarcinogenesis in rats. J. Cell. Biochem..

[93] Chakraborty T., Chatterjee A., Dhachinamoorthi D., Srivastawa S., Panayappan L., Chatterjee M. (2006). Vanadium limits the expression of proliferating cell nuclear antigen and inhibits early DNA damage during diethylnitrosamine-induced hepatocellular preneoplasia in rats. Environ. Mol. Mutagen..

[94] Chakraborty T., Chatterjee A., Rana A., Dhachinamoorthi D., Kumar P.A., Chatterjee M. (2007). Carcinogen-induced early molecular events and its implication in the initiation of chemical hepatocarcinogenesis in rats: Chemopreventive role of vanadium on this process. Biochim. Biophys. Acta.

[95] Chakraborty T., Chatterjee A., Saralaya M.G., Chatterjee M. (2006). Chemopreventive effect of vanadium in a rodent model of chemical hepatocarcinogenesis: Reflections in oxidative DNA damage, energy-dispersive X-ray fluorescence profile and metallothionein expression. J. Biol. Inorg. Chem..

[96] Chakraborty T., Pandey N., Chatterjee A., Ghosh B., Rana B., Chatterjee M. (2006). Molecular basis of anticlastogenic potential of vanadium in vivo during the early stages of diethylnitrosamine-induced hepatocarcinogenesis in rats. Mutat. Res..

[97] Bishayee A., Roy S., Chatterjee M. (1999). Characterization of selective induction and alteration of xenobiotic biotransforming enzymes by vanadium during diethylnitrosamine-induced chemical rat liver carcinogenesis. Oncol. Res..

[98] Chakraborty T., Chatterjee A., Rana A., Rana B., Palanisamy A., Madhappan R., Chatterjee M. (2007). Suppression of early stages of neoplastic transformation in a two-stage chemical hepatocarcinogenesis model: Supplementation of vanadium, a dietary micronutrient, limits cell proliferation and inhibits the formations of 8-hydroxy-2′-deoxyguanosines and DNA strand-breaks in the liver of sprague-dawley rats. Nutr. Cancer.

[99] Chakraborty T., Ghosh S., Datta S., Chakraborty P., Chatterjee M. (2003). Vanadium suppresses sister-chromatid exchange and DNA-protein crosslink formation and restores antioxidant status and hepatocellular architecture during 2-acetylaminofluorene-induced experimental rat hepatocarcinogenesis. J. Exp. Ther. Oncol..

[100] Chakraborty T., Samanta S., Ghosh B., Thirumoorthy N., Chatterjee M. (2005). Vanadium induces apoptosis and modulates the expressions of metallothionein, Ki-67 nuclear antigen, and p53 during 2-acetylaminofluorene-induced rat liver preneoplasia. J. Cell. Biochem..

[101] Samanta S., Chatterjee M., Ghosh B., Rajkumar M., Rana A. (2008). Vanadium and 1, 25 (OH)_2_ vitamin D 3 combination in inhibitions of 1,2, dimethylhydrazine-induced rat colon carcinogenesis. Biochim. Biophys. Acta.

[102] Samanta S., Swamy V., Suresh D., Rajkumar M., Rana B., Rana A., Chatterjee M. (2008). Protective effects of vanadium against dmh-induced genotoxicity and carcinogenesis in rat colon: Removal of O^6^-methylguanine DNA adducts, p53 expression, inducible nitric oxide synthase downregulation and apoptotic induction. Mutat. Res..

[103] Kanna P.S., Mahendrakumar C.B., Indira B.N., Srivastawa S., Kalaiselvi K., Elayaraja T., Chatterjee M. (2004). Chemopreventive effects of vanadium toward 1,2-dimethylhydrazine-induced genotoxicity and preneoplastic lesions in rat colon. Environ. Mol. Mutagen..

[104] Thompson H.J., Chasteen N.D., Meeker L.D. (1984). Dietary vanadyl(IV) sulfate inhibits chemically-induced mammary carcinogenesis. Carcinogenesis.

[105] Sankar Ray R., Roy S., Ghosh S., Kumar M., Chatterjee M. (2004). Suppression of cell proliferation, DNA protein cross-links, and induction of apoptosis by vanadium in chemical rat mammary carcinogenesis. Biochim. Biophys. Acta.

[106] Sankar Ray R., Roy S., Samanta S., Maitra D., Chatterjee M. (2005). Protective role of vanadium on the early process of rat mammary carcinogenesis by influencing expression of metallothionein, ggt-positive foci and DNA fragmentation. Cell Biochem. Funct..

[107] Ray R.S., Basu M., Ghosh B., Samanta K., Chatterjee M. (2005). Vanadium, a versatile biochemical effector in chemical rat mammary carcinogenesis. Nutr. Cancer.

[108] Manna S., Das S., Chatterjee M., Janarthan M. (2011). Combined supplementation of vanadium and fish oil suppresses tumor growth, cell proliferation and induces apoptosis in dmba-induced rat mammary carcinogenesis. J. Cell. Biochem..

[109] Bishayee A., Oinam S., Basu M., Chatterjee M. (2000). Vanadium chemoprevention of 7,12-dimethylbenz(a)anthracene-induced rat mammary carcinogenesis: Probable involvement of representative hepatic phase i and ii xenobiotic metabolizing enzymes. Breast Cancer Res. Treat..

[110] Köpf-Maier P., Wagner W., Hesse B., Köpf H. (1981). Tumor inhibition by metallocenes: Activity against leukemias and detection of the systemic effect. Eur. J. Cancer.

[111] Bishayee A., Waghray A., Patel M.A., Chatterjee M. (2010). Vanadium in the detection, prevention and treatment of cancer: The in vivo evidence. Cancer Lett..

[112] Zhai F., Wang X., Li D., Zhang H., Li R., Song L. (2009). Synthesis and biological evaluation of decavanadate na4co(h2o)6v10o28.18h2o. Biomed. Pharmacother..

[113] Galani A., Tsitsias V., Stellas D., Psycharis V., Raptopoulou C.P., Karaliota A. (2015). Two novel compounds of vanadium and molybdenum with carnitine exhibiting potential pharmacological use. J. Inorg. Biochem..

[114] Cantley L.C., Josephson L., Warner R., Yanagisawa M., Lechene C., Guidotti G. (1977). Vanadate is a potent (Na,K)-ATPase inhibitor found in ATP derived from muscle. J. Biol. Chem..

[115] Collauto A., Mishra S., Litvinov A., Mchaourab H.S., Goldfarb D. (2017). Direct spectroscopic detection of atp turnover reveals mechanistic divergence of abc exporters. Structure.

[116] Willsky G.R., White D.A., McCabe B.C. (1984). Metabolism of added orthovanadate to vanadyl and high-molecular-weight vanadates by saccharomyces cerevisiae. J. Biol. Chem..

[117] Harding M.M., Mokdsi G. (2000). Antitumour metallocenes: Structure-activity studies and interactions with biomolecules. Curr. Med. Chem..

[118] Köpf-Maier P., Wagner W., Liss E. (1983). Induction of cell arrest at G1/S and in G2 after treatment of ehrlich ascites tumor cells with metallocene dichlorides and cis-platinum in vitro. J. Cancer Res. Clin. Oncol..

[119] Rehder D. (2013). Vanadium. Its role for humans. Met. Ions Life Sci.

[120] Korbecki J., Baranowska-Bosiacka I., Gutowska I., Chlubek D. (2015). Vanadium compounds as pro-inflammatory agents: Effects on cyclooxygenases. Int. J. Mol. Sci..

[121] Ress N.B., Chou B.J., Renne R.A., Dill J.A., Miller R.A., Roycroft J.H., Hailey J.R., Haseman J.K., Bucher J.R. (2003). Carcinogenicity of inhaled vanadium pentoxide in F344/N rats and B6C3F1 mice. Toxicol. Sci..

[122] Boulassel B., Sadeg N., Roussel O., Perrin M., Belhadj-Tahar H. (2011). Fatal poisoning by vanadium. Forensic Sci. Int..

[123] Liu Y., Jie X., Guo Y., Zhang X., Wang J., Xue C. (2016). Green synthesis of oxovanadium(IV)/chitosan nanocomposites and its ameliorative effect on hyperglycemia, insulin resistance, and oxidative stress. Biol. Trace Elem. Res..

[124] Lichawska M.E., Bodek K.H., Jezierska J., Kufelnicki A. (2014). Coordinative interaction of microcrystalline chitosan with oxovanadium (IV) ions in aqueous solution. Chem. Cent. J..

[125] Kremer L.E., McLeod A.I., Aitken J.B., Levina A., Lay P.A. (2015). Vanadium(V) and -(IV) complexes of anionic polysaccharides: Controlled release pharmaceutical formulations and models of vanadium biotransformation products. J. Inorg. Biochem..

[126] Chen Y., Cheng L., Dong Z., Chao Y., Lei H., Zhao H., Wang J., Liu Z. (2017). Degradable vanadium disulfide nanostructures with unique optical and magnetic functions for cancer theranostics. Angew. Chem. Int. Ed. Engl..

[127] Matsumura Y., Maeda H. (1986). A new concept for macromolecular therapeutics in cancer chemotherapy: Mechanism of tumoritropic accumulation of proteins and the antitumor agent smancs. Cancer Res..

[128] Torchilin V.P. (2007). Targeted pharmaceutical nanocarriers for cancer therapy and imaging. AAPS J..

[129] Ivankovic S., Music S., Gotic M., Ljubesic N. (2006). Cytotoxicity of nanosize V_2_O_5_ particles to selected fibroblast and tumor cells. Toxicol. In Vitro.

